# Supplementary material for: Universality of political corruption networks
Source: Sci Rep. 2022 Apr 27;12:6858. doi: 10.1038/s41598-022-10909-2 (PMC9046414; doi:10.1038/s41598-022-10909-2)
Supplement: Supplementary file 1 — Supplementary Figures. [file 41598_2022_10909_MOESM1_ESM.pdf]

Universality of political corruption networks

Alvaro F. Martins[1], Bruno R. da Cunha[2,3], Quentin S. Hanley[4], Sebastián Gonçalves[5], Matjaž Perc[6,7,8,9,*], and Haroldo V. Ribeiro[1]

[1]Departamento de Física, Universidade Estadual de Maringá – Maringá, PR 87020-900, Brazil
[2]Rio Grande do Sul Superintendency, Brazilian Federal Police – Porto Alegre, RS 90160-093, Brazil
[3]National Police Academy, Brazilian Federal Police – Brasília, DF 71559-900, Brazil
[4]School of Science and Technology, Nottingham Trent University, Clifton Lane, Nottingham NG11 8NS, United Kingdom
[5]Instituto de Física, Universidade Federal do Rio Grande do Sul – Porto Alegre, RS 91501-970, Brazil
[6]Faculty of Natural Sciences and Mathematics, University of Maribor, Koroška cesta 160, 2000 Maribor, Slovenia
[7]Department of Medical Research, China Medical University Hospital, China Medical University, Taichung, Taiwan
[8]Alma Mater Europaea, Slovenska ulica 17, 2000 Maribor, Slovenia
[9]Complexity Science Hub Vienna, Josefstädterstraße 39, 1080 Vienna, Austria
[*]email: matjaz.perc@gmail.com

Supplemental Materials

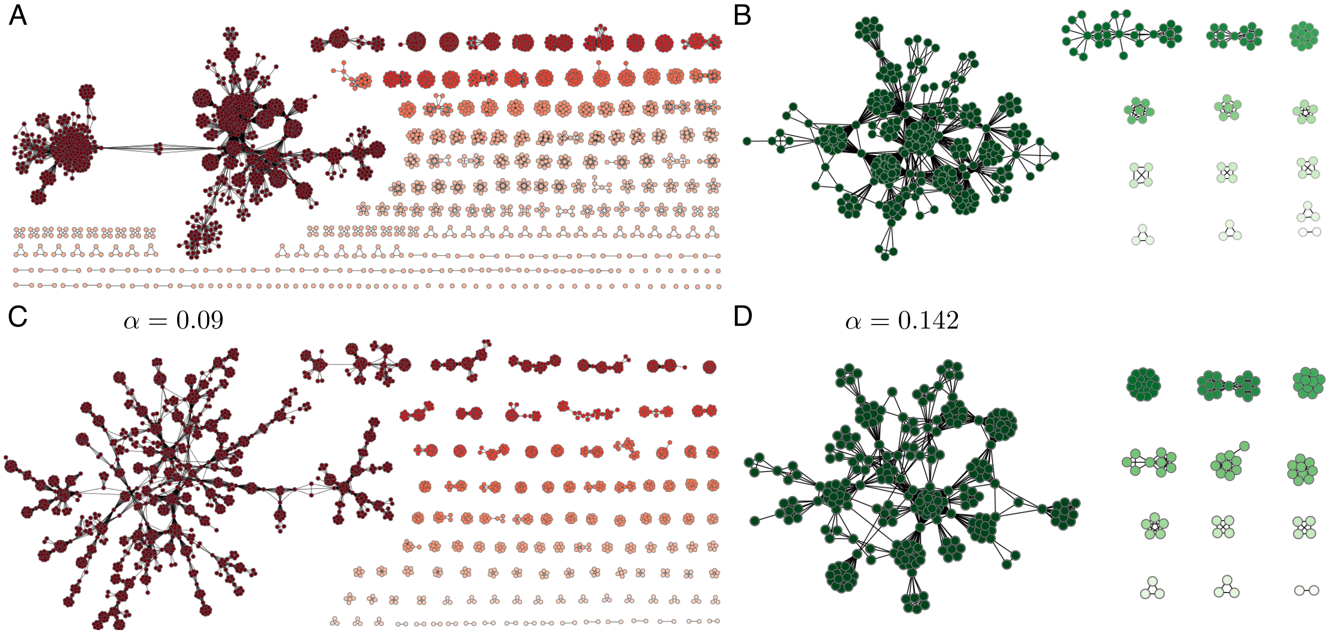

Figure S1. Visual comparison between simulated and empirical corruption networks. The Spanish and Brazilian corruption networks (panels A and B, respectively) compared to typical networks generated by our model using the empirical values of the recidivism rate of Spain ($\alpha = 0.09$, panel C) and Brazil ($\alpha = 0.14$, panel D). Both simulated networks have been generated using the exact number of political scandals of the empirical data. Node color stands for network components.

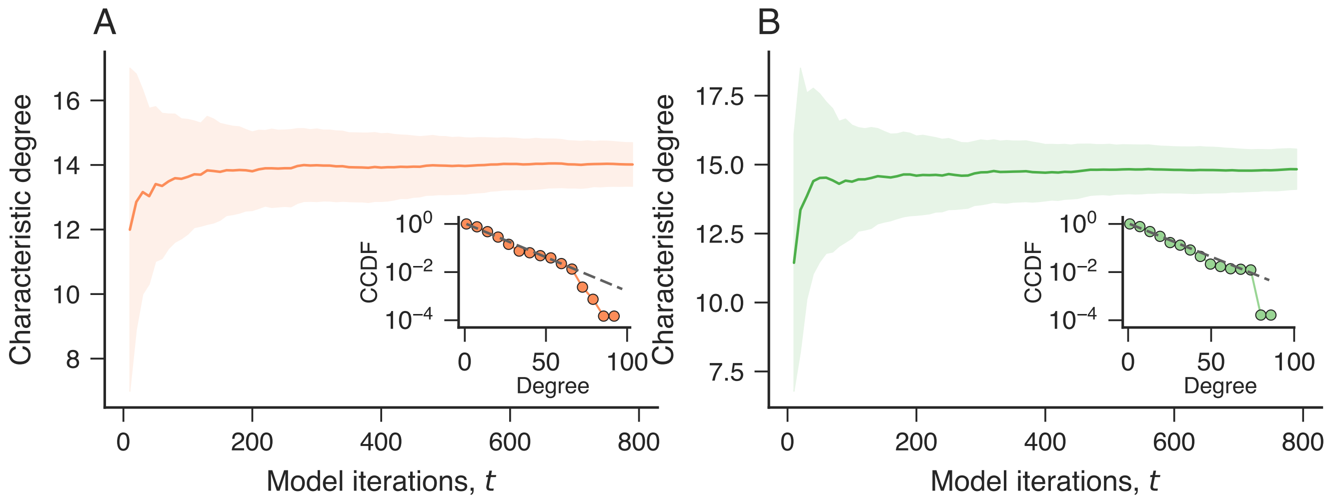

Figure S2. Our model replicates the exponential degree distribution and the approach to a constant value of the characteristic degree observed in empirical networks. (A) Average value of the maximum-likelihood estimate of the characteristic degree obtained from one hundred simulations using the Spanish recidivism rate ($\alpha = 0.09$) as a function of the number of model iterations t (that is, number of scandals added to the network). The shaded region stands for 95% bootstrap confidence intervals. The inset shows the complementary cumulative degree distribution after $t = 800$ model iterations, where the dashed line indicates the exponential distribution adjusted to data. Panel (B) shows the same quantities for simulations using the Brazilian recidivism rate ($\alpha = 0.14$).

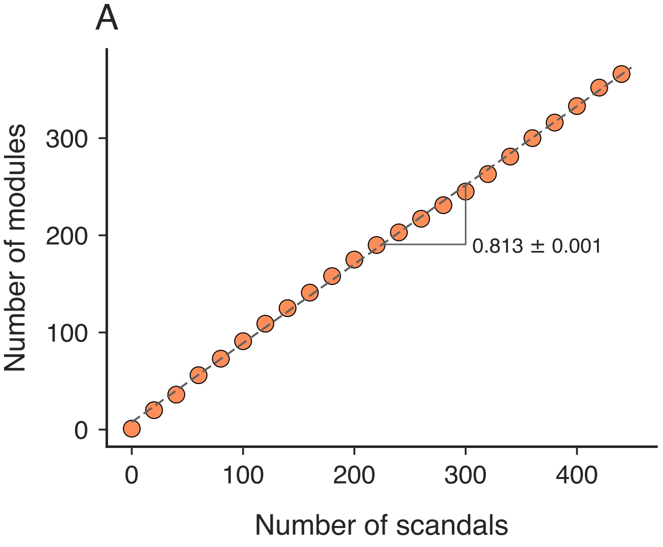

Figure S3. Our model replicates the linear association between the number of network modules and the number of scandals observed during the growth of empirical networks. Panels (A) and (B) show the average association between the number of network modules and the total of scandals estimated from one hundred simulations with the Spanish and Brazilian recidivism rates, respectively. In both panels, the dashed lines represent linear models adjusted to data and the values within these panels indicate the average ratio between network modules and scandals during the growth of simulated networks.

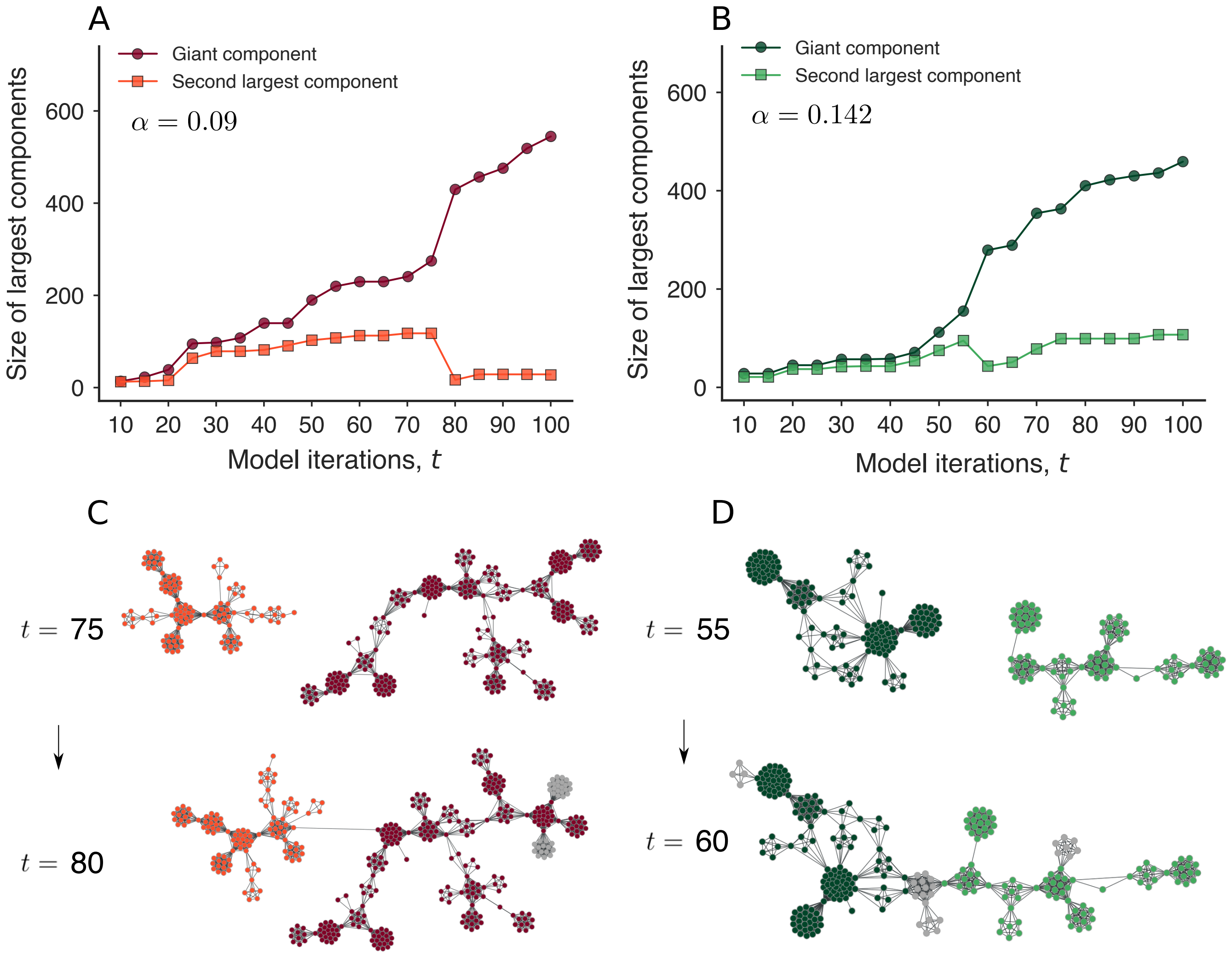

Figure S4. Our model replicates the coalescence of network components observed during the growth of empirical corruption networks. Panels (A) and (B) depict the evolution of the size of the giant component (circle) and second-largest component (square) of typical simulated networks using the Spanish and Brazilian recidivism rates, respectively. Similarly to the empirical networks, we observe that the simulated behavior exhibits abrupt changes caused by the emergence of new scandals involving recidivist agents. Panels (C) and (D) show snapshot visualizations of simulated networks before and after the abrupt changes observed in panels (A) and (B), respectively. In these snapshots, new nodes are colored in gray.

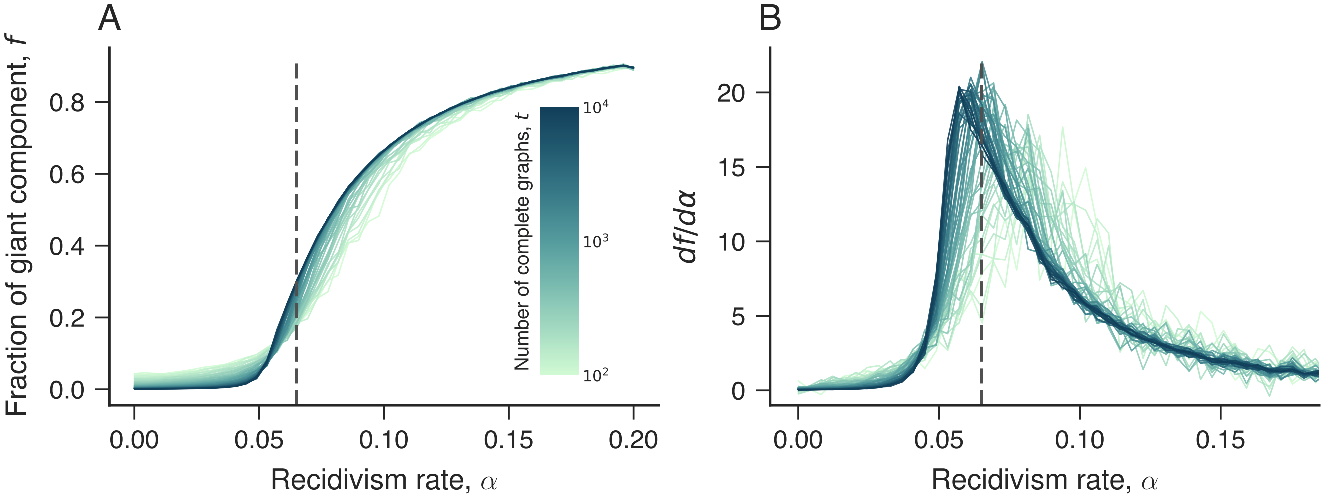

Figure S5. Model behavior near the criticality for different network sizes. (A) Average fraction of the giant component of simulated networks f as a function of the recidivism rate α. (B) The derivative of f with respect to α. In both panels, the different curves represent the average model behavior over one hundred realizations (with $s_c = 7$, $\beta = 12$, and $p = 0.025$) and after including t complete graphs (the value of t is indicated by the color code). The dashed vertical line indicates the critical recidivism rate. We observe that the transition does not become more abrupt when increasing the network size, suggesting that our model displays a continuous (second-order) phase transition.

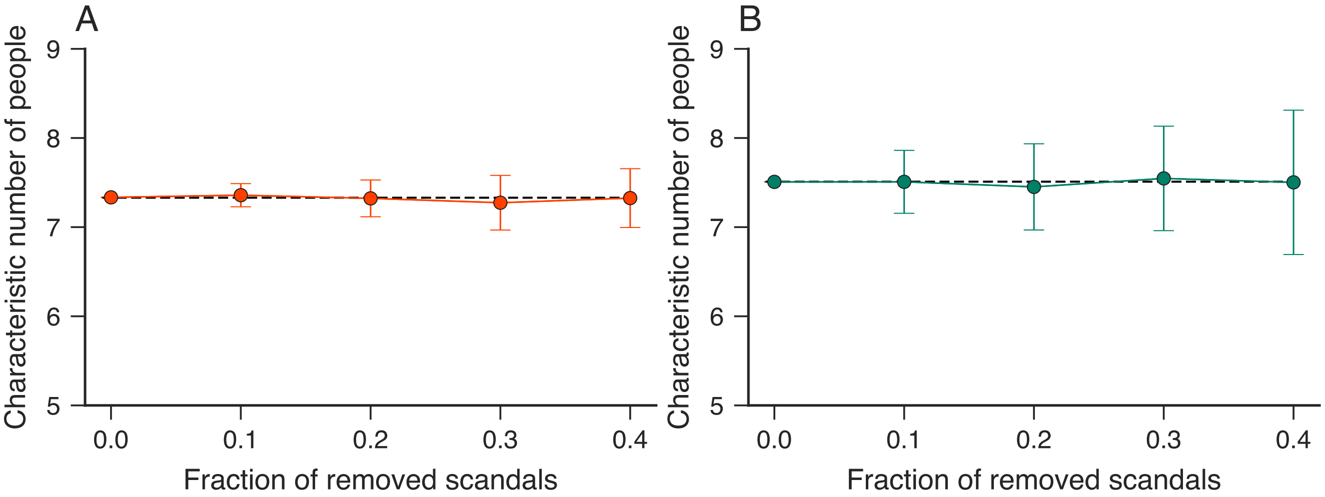

Figure S6. Robustness of the size distribution of scandals against randomly removing a fraction of scandals from our datasets. Estimates of the characteristic number of people involved in political corruption scandals in (A) Spain and (B) Brazil as a function of the fraction of removed scandals. The dashed lines indicate the values when considering all scandals in our datasets. The markers represent average values estimated from one hundred realizations of the randomly removing process, and the error bars stand for one standard deviation of these values. In both cases, the characteristic number of people remains very stable even after removing 40% of the scandals in our datasets.

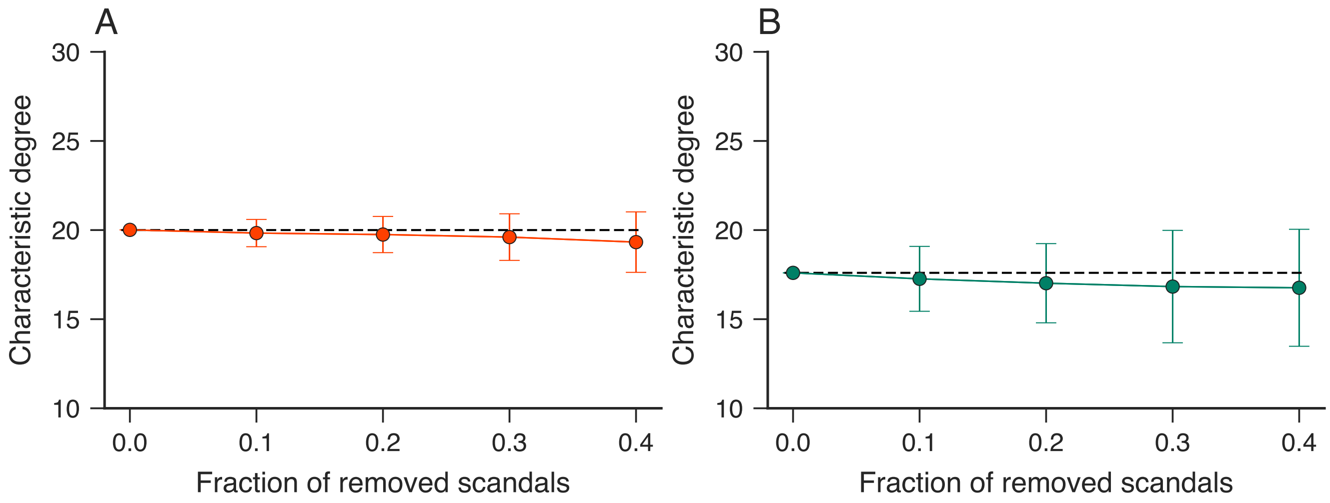

Figure S7. Robustness of the degree distribution of corruption networks against randomly removing a fraction of scandals from our datasets. Estimates of characteristic degree for the latest stage of the (A) Spanish and (B) Brazilian corruption networks as a function of the fraction of removed scandals. The dashed lines indicate the values when considering all scandals in our datasets. The markers represent average values estimated from one hundred realizations of the randomly removing process, and the error bars stand for one standard deviation of these values. In both cases, the characteristic degree remains very stable even after removing 40% of the scandals in our datasets.

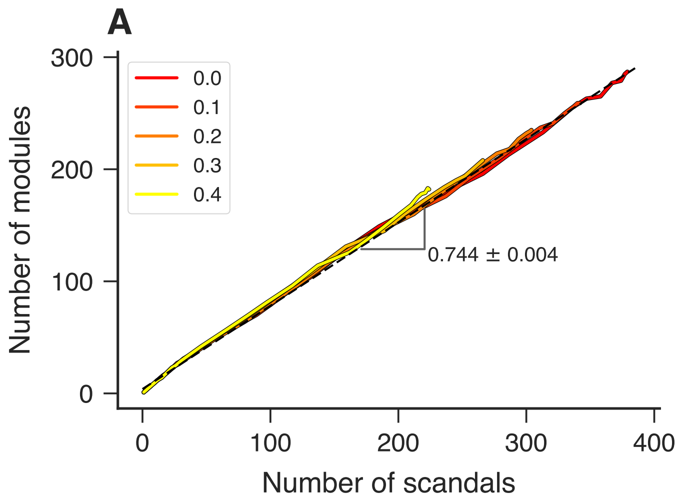

Figure S8. Robustness of the association between the number of network modules and the total of political scandals against randomly removing a fraction of scandals from our datasets. The curves show the relationships between the number of modules and the number of scandals for each year of the (A) Spanish and (B) Brazilian networks under different fractions of removed scandals (indicated by the color code). The dashed lines represent a linear model adjusted to the relationship obtained when considering all scandals in our datasets. We note the linear association remains a good approximation for the empirical behavior even after removing 40% of the scandals from our datasets.

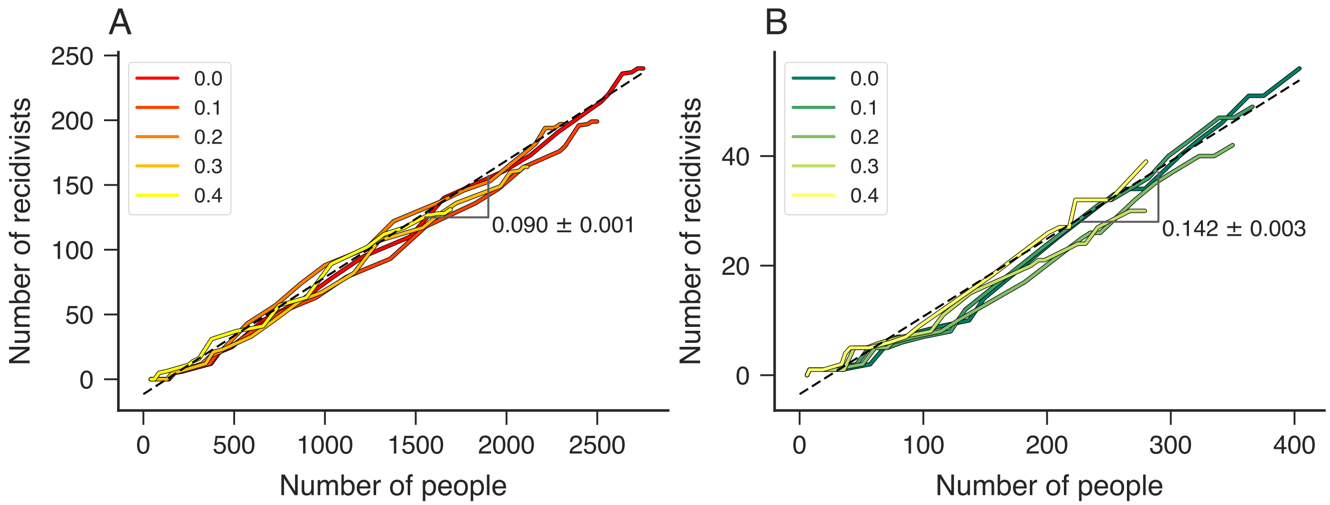

Figure S9. Robustness of the association between the number of recidivist agents and the total number of people against randomly removing a fraction of scandals from our datasets. The curves show the relationships between the number of recidivist agents and the total number of people for each year of the (C) Spanish and (D) Brazilian corruption networks under different fractions of removed scandals (indicated by the color code). The dashed lines represent a linear model adjusted to the relationship obtained when considering all scandals in our datasets. We note that linear association remains a good approximation for the empirical behavior even after removing 40% of the scandals in our datasets.
